# Intensive care unit patients’ opinion on enrollment in clinical research: A multicenter survey

**DOI:** 10.1371/journal.pone.0236675

**Published:** 2020-08-13

**Authors:** Flavia Julie do Amaral Pfeilsticker, Carolina Aguiar Sant Anna Siqueri, Niklas Soderberg Campos, Fernanda Guimarães Aguiar, Maria Laura Romagnoli, Renato Carneiro de Freitas Chaves, Carolina Scoqui Guimarães, Adriano José Pereira, Ricardo Luiz Cordioli, Ary Serpa Neto, Murillo Santucci Cesar Assuncão, Thiago Domingos Corrêa

**Affiliations:** 1 Department of Critical Care Medicine, Hospital Israelita Albert Einstein, São Paulo, Brazil; 2 Intensive Care Unit, Hospital Municipal Vila Santa Catarina, São Paulo, Brazil; Azienda Ospedaliero Universitaria Careggi, ITALY

## Abstract

**Background:**

In most emergency situations or severe illness, patients are unable to consent for clinical trial enrollment. In such circumstances, the decision about whether to participate in a scientific study or not is made by a legally designated representative.

**Objective:**

To address the willingness of patients admitted to the intensive care unit (ICU) to be enrolled in a scientific study as volunteers, and to assess the agreement between patients’ and their legal representatives’ opinion concerning enrollment in a scientific study.

**Methods:**

This survey was conducted in two hospitals in São Paulo, Brazil. Patients (≥18 years) with preserved cognitive functions accompanied by a surrogate admitted to the ICU were eligible for this study. A survey containing 28 questions for patients and 8 questions for surrogates was applied within the first 48h from ICU admission. The survey for patients comprised three sections: demographic characteristics, opinion about participation in clinical research and knowledge about the importance of research. The survey for legal representatives contained two sections: demographic characteristics and assessment of legal representatives’ opinion in authorizing patients to be enrolled in research.

**Results:**

Between January 2017 and May 2018, 208 pairs of ICU patients and their respective legal representatives answered the survey. Out of 208 ICU patients answering the survey, 73.6% (153/208) were willing to be enrolled in the study as volunteers. Of those patients, 65.1% (97/149) would continue participating in a research even if their legal representative did not support their enrollment. Agreement between patients’ and surrogates’ opinion concerning participation was poor [Kappa = 0.11 (IC95% -0.02 to 0.25)]. If a consent for study participation had been obtained, 69.1% (103/149) of patients would continue participating in the study until its conclusion, and 23.5% (35/149) would allow researchers to use data collected to date, but would withdraw from the study on that occasion.

**Conclusion:**

The majority of patients admitted to the ICU were willing to be enrolled in a scientific study as volunteers, also after a deferred informed consent procedure has been used. Nevertheless, contradictory opinions between patients and their and their legal representatives’ concerning enrollment in a scientific study were often observed.

## Introduction

Scientific research involving human beings has been the cornerstone of the development of medical knowledge. Nevertheless, in order to preserve the principle of autonomy of research subjects as stated in Helsinki Declaration [1], study investigators must guarantee that an informed consent is obtained from each study participant before enrollment [2]. However, life-threatening conditions, commonly observed among patients admitted to the intensive care unit (ICU), may preclude ICU patients from consenting enrollment in a clinical trial [3, 4]. In such circumstances, consent must be obtained from a legally authorized representative [1].

The acquisition of consent by proxies can be challenging in the critical care setting. In many circumstances during the ICU stay, legally authorized representatives may not be readily available or may not even exist, precluding conduction of a clinical trial in emergency situations with a narrow therapeutic window [2]. Thus, depending on international and local ethics committees’ policies, consent can be waived or deferred by patient (i.e., patient deferred consent) or by proxy (i.e., proxy deferred consent) [3]. When a deferred consent is obtained, the patient is enrolled in the study but the informed consent must be obtained as soon as possible by patient or his/her legal representative [3].

The discussion regarding the use of deferred proxy consent instead of regular consent is reasonable, considering that surrogates’ decision may not reflect patients’ opinion [5]. Moreover, critical care conditions may trigger emotional stress disorders [6], religious concerns [7] and financial problems [8], which commonly affect relatives’ decision making. Despite the relevance of this topic, Brazil is lacking in studies addressing willingness of patients admitted to the ICU to be enrolled in a scientific study as volunteers. Moreover, agreements between ICU patients’ and their legal representatives’ opinion concerning enrollment in a scientific study is unknown.

## Objectives

Our purpose was to address the willingness of patients admitted to the ICU to be enrolled in a scientific study as volunteers, and to assess the agreement between ICU patients’ and their legal representatives’ opinion concerning enrollment in a scientific study.

## Material and methods

### Study design and settings

This prospective observational study was conducted in two ICUs located in two hospitals (private and public) in São Paulo, Brazil. The Ethics Committee of Hospital Israelita Albert Einstein (CAAE: 62100416.8.1001.0071) and of Secretaria Municipal da Saúde de São Paulo–SMS/SP (Municipal Secretary of Health of São Paulo) (CAAE: 62100416.8.3001.0086) approved the study protocol. Written informed consent was obtained from each study participant. This study was reported in accordance with The Strengthening the Reporting of Observational Studies in Epidemiology (STROBE) guidelines [9].

### Characteristics of study participants

Adult patients (≥18 years old) with preserved cognitive function accompanied by a surrogate admitted to the ICU were eligible for this study. Patients under 18 years old, patients unable to understand or to speak Portuguese, patients with no legal representative during ICU stay, patients with impaired consciousness at the time of evaluation (i.e., sedated patients or patients with delirium), and patients with developmental delay were excluded.

### The survey

The questionnaire was developed by the authors (FJAP, RLC and TDC). Content validation was performed by a panel of senior researchers (AJP, ASN and MSCA). They critically revised the survey instrument and judged whether the instrument meets its goal. The panel of senior researchers sent the survey for the developers to make the necessary adjustments. These adjustments were performed and the revised survey was reassessed by the panel. After the third revision, content validity was supported by the panel. Face validity, i.e, whether the survey instrument appears to test what it is supposed to, and questionnaire's psychometric properties were tested by all other co-authors and by a small group of volunteers (10 employers) in both ICUs.

The survey for patients comprised 28 questions distributed in three sections—demographic characteristics, opinion about participation in clinical research and knowledge of the importance of research, respectively (Supporting Information). The survey for legal representatives contained 8 questions distributed in two sections—demographic characteristics and assessment of the legal representatives’ opinion on authorizing patients to be enrolled in research (Additional file 1).

Participants who accepted to participate in this study responded to a traditional paper and pencil questionnaire, which was then put into an opaque envelope to preserve their anonymity. The survey was simultaneously applied to patients and their legal representative within the first 48 hours of ICU admission. Once responded, the answers were not able to be revised. The study database was structured in the Research Electronic Data Capture (REDCap) [10], hosted in a private and safe server at Hospital Israelita Albert Einstein, which was filled out by the investigators. No financial incentives were offered for participants of this study. A threshold of more than 90% of answers was used to determine completion of questionnaires.

### Data analysis

To achieve 95% confidence and 5% precision by adopting a conservative sample (50% of the participants answering YES to the question “Would you participate in a clinical trial as a volunteer?”), an estimated sample size of 208 pairs of patients and legal representatives was determined.

Participants were pooled into two groups according to their willingness to participate as volunteers in a scientific study (Yes/Probably yes) and (No/Probably no) and accordingly to the type of hospital (Private vs. Public). Categorical variables were displayed as absolute and relative frequencies. Numerical variables were presented as mean and standard deviation (SD) or median with interquartile range (IQR) in case of non-normal distribution, tested with Kolmogorov-Smirnov test.

Comparisons were performed between the pooled groups (Yes/Probably yes) and (No/Probably no) and accordingly to the type of hospital (Private vs. Public). Categorical variables were compared with chi-square test. Continuous variables were compared using independent t test or Mann-Whitney U test in case of non-normal distribution, tested by the Kolmogorov-Smirnov test. Agreement between patients and their legal representatives was assessed using kappa statistics. Kappa value closer to zero should be interpreted as no agreement, whereas Kappa value close to one should be interpreted as perfect agreement.

A multivariable logistic regression analysis was performed to address patients’ characteristics associated with their willingness to participate as volunteers in a scientific study. Variables considered for the multivariate modeling included age, gender, educational level, religion and family income. Results were presented as odds ratio (OR) with 95% confidence interval (95%CI). The performance of the model was evaluated by assessing discrimination and calibration. Discrimination was evaluated with the area under the receiver operating characteristics curve (AUROC) and calibration was evaluated with the Hosmer-Lemeshow test.

Two-tailed tests were used and when p<0.05, the test was considered statistically significant. No adjustment was made for missing data. The SPSS™ (IBM™ Statistical Package for the Social Science version 23.0) was used for statistical analyses and GraphPad Prism version 7.0 (GraphPad Software, California, USA) was used for graph plotting.

## Results

### Participants’ characteristics

Between January 2017 and May 2018, 208 pairs of ICU patients and their respective legal representatives answered the survey [87.0% (181/208) private hospital and 13.0% (27/208) public hospital]. The median (IQR) age of patients was 60 (43–75) years, 49% were female and 56.7% had a high educational level. The median (IQR) age of legal representatives was 60 (43–75) years, 65.9% were female and 68.8% were next of kin (spouse/son/daughter). The characteristics of study participants according to their willingness to participate as volunteers in a scientific study and according to the type of hospital (private vs. public) are presented, respectively, in Table 1 and S1 Table in S1 File.

**Table 1:** Characteristics of study participants according to their willingness to participate as volunteers in a scientific study. Data presented as median (interquartile range) or nº/total (%)^#^.

Characteristics	All patients (N = 208)	Yes / Probably yes (N = 153)	No / Probably no (N = 55)	P value
Age, years	60 (43–75)	58 (43–70)	70 (46–82)	0.002^a^
Female, gender	102/208 (49.0)	69/153 (45.1)	33/55 (60.0)	0.058^b^
Educational level				0.803^b^
Master’s / PhD	21/208 (10.1)	17/153 (11.1)	4/55 (7.3)	
Higher education	118/208 (56.7)	85/153 (55.6)	33/55 (60.0)	
High school	40/208 (19.2)	30/153 (19.6)	10/55 (18.2)	
Primary school	27/208 (13.0)	19/153 (12.4)	8/55 (14.5)	
Illiterate	2/208 (1.0)	2/153 (1.3)	0/55 (0.0)	
Religion				0.013^b^
Catholic	145/208 (69.7)	106/153 (69.3)	39/55 (70.9)	
Other	20/208 (9.6)	15/153 (9.8)	5/55 (9.1)	
Evangelic	15/208 (7.2)	13/153 (8.5)	2/55 (3.6)	
No religion	15/208 (7.2)	14/153 (9.2)	1/55 (1.8)	
Jewish	13/208 (6.3)	5/153 (3.3)	8/55 (14.5)	
Place of residence				0.972^b^
Southeast	173/208 (83.2)	127/153 (83.0)	46/55 (83.6)	
South	16/208 (7.7)	11/153 (7.2)	5/55 (9.1)	
Central-west	10/208 (4.8)	8/153 (5.2)	2/55 (3.6)	
Northeast	5/208 (2.4)	4/153 (2.6)	1/55 (1.8)	
North	4/208 (1.9)	3/153 (2.0)	1/55 (1.8)	
Family income				0.708^b^
>10 minimum wages	133/198 (67.2)	97/146 (66.4)	36/52 (69.2)	
6–10 minimum wages	24/198 (12.1)	16/146 (11.0)	8/52 (15.4)	
2–5 minimum wages	33/198 (16.7)	27/146 (18.5)	6/52 (11.5)	
1 minimum wage	7/198 (3.5)	5/146 (3.4)	2/52 (3.8)	
No income	1/198 (0.5)	1/146 (0.7)	0/52 (0.0)	
**Legal Representatives**				
Age, years	49 (37–60)	48 (37–61)	50 (38–58)	0.781^a^
Female, gender	137 (65.9)	104/153 (68.0)	33/55 (60.0)	0.285^b^
Kinship degree				0.328^b^
Husband / wife	75 (36.1)	57/153 (37.3)	18/55 (32.7)	
Son / daughter	68 (32.7)	48/153 (31.4)	20/55 (36.4)	
Parent	25 (12.0)	21/153 (13.7)	4/55 (7.3)	
Brother / sister	15 (7.2)	12/153 (7.8)	3/55 (5.5)	
Other	25 (12.0)	15/153 (9.8)	10/55 (18.2)	

#: For variables with missing data, summary data are based on available cases. P values were calculated with (a): Mann-Whitney U test or (b) chi-square test.

### Patients’ opinion about participation in clinical research

**Fig 1:**
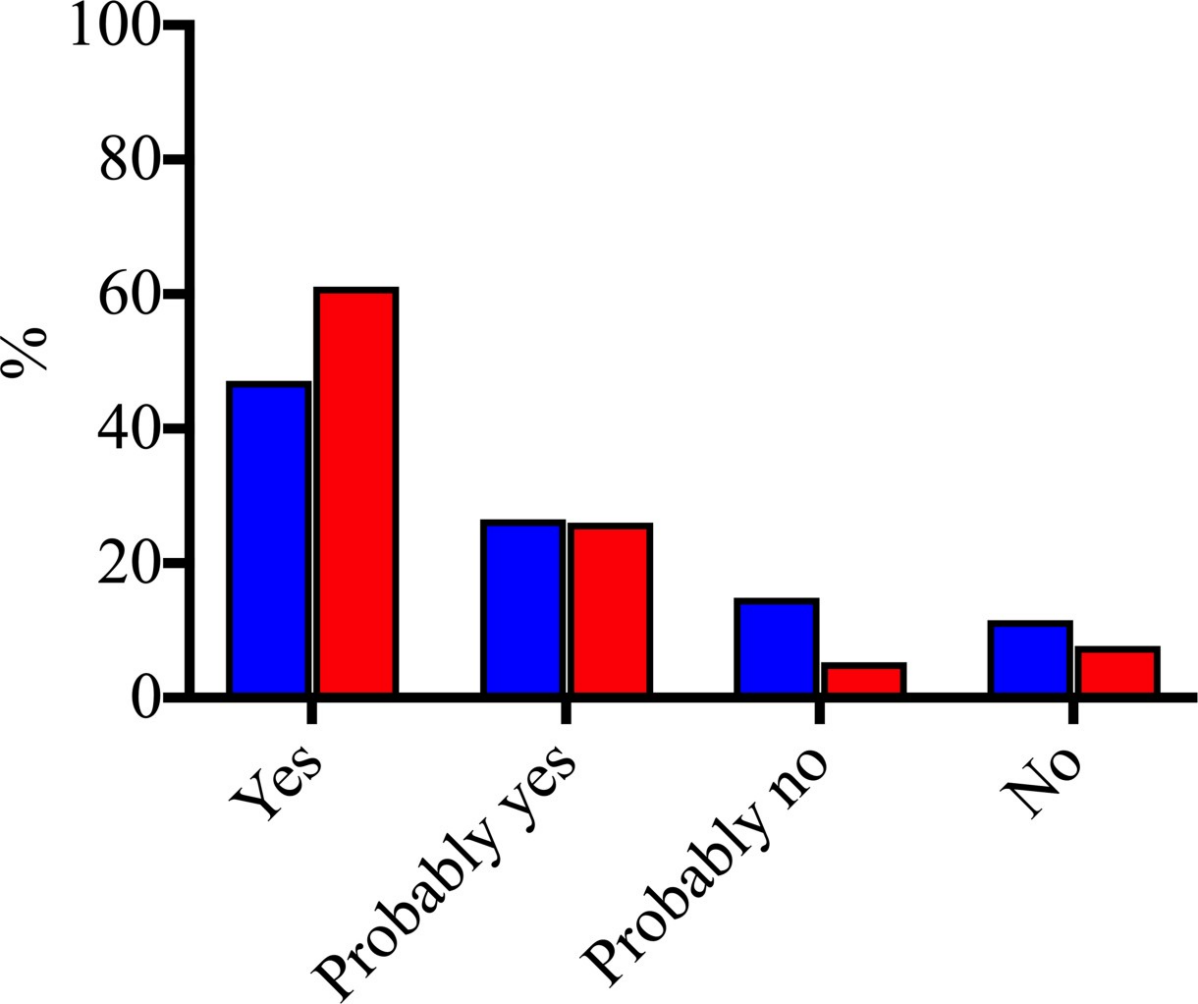
Patients’ and legal representatives’ answers to the respective questions “Would you like to be enrolled in a scientific study as a volunteer?” and “Would you authorize your relative to participate as a volunteer in scientific research during their stay in the ICU?”. Blue bars represent patients and red bars represent legal representatives.

Out of 208 ICU patients who answered the survey, 73.6% (153/208) were willing to be enrolled in a scientific study as volunteers (Fig 1 and S2 Table in S1 File). Approximately 14.0% of patients [13.9% (29/208)] who answered the survey had never been enrolled in a scientific study. The willingness to be enrolled in a scientific study as volunteers did not differ between patients that had already been enrolled in a scientific study as a volunteer compared to those patients that had never been enrolled in a scientific study [86.2% (25/29) vs. 71.5% (128/179), respectively, p = 0.096].

Less than one third of the patients [28.0% (42/150)] that were willing to be enrolled in a scientific study as a volunteer had ever heard of informed consent. The most common reason given by patients for choosing to be volunteers in a scientific study was the fact they believed the study results would bring benefits to the general population in the future [88.2% (135/153) of patients] (Table 2).

**Table 2:** Patients willing (Yes / Probably yes) to be enrolled in a scientific study opinion about participation in clinical research accordingly to the type of hospital. Data presented nº/total (%)^#^.

	All patients (N = 153)	Private Hospital (N = 130)	Public Hospital (N = 23)	P value
Have you ever been enrolled in a scientific study?				0.021
Yes	25/153 (16.3)	25/130 (19.2)	0/23 (0.0)	
No	128/153 (83.7)	105/130 (80.8)	23/23 (100.0)	
What motivates you to be a volunteer in a scientific study?				
The study offers benefits for the general population in the future	135/153 (88.2)	114/130 (87.7)	21/23 (91.3)	0.743
The study offers benefits to you immediately/in the future	101/153 (66.0)	88/130 (67.7)	13/23 (56.5)	0.342
Your physician’s request	93/153 (60.8)	79/130 (60.8)	14/23 (60.9)	1.000
No treatment options available	100/153 (65.3)	89/130 (68.5)	11/23 (47.8)	0.062
The study offers financial gains for your participation	24/153 (15.7)	19/130 (14.6)	5/23 (21.7)	0.533
Would you change your opinion, i.e., no longer accept to participate in a scientific study if your legal representative did not support your enrollment?				
Yes	34/149 (22.8)	29/127 (22.8)	5/22 (22.7)	0.670
Probably yes	18/149 (12.1)	16/127 (12.6)	2/22 (9.1)	
Probably no	25/149 (16.8)	23/127 (18.1)	2/22 (9.1)	
No	72/149 (48.3)	59/127 (46.5)	13/22 (59.1)	
Would you change your opinion, i.e., no longer accept to participate in a scientific study if your primary attending physician did not support your enrollment?				0.892
Yes	75/150 (50.0)	65/128 (50.8)	10/22 (45.5)	
Probably yes	28/150 (18.7)	23/128 (18.0)	5/22 (22.7)	
Probably no	16/150 (10.6)	13/128 (10.2)	3/22 (13.6)	
No	31/150 (20.7)	27/128 (21.1)	4/22 (18.2)	
Would you participate as a volunteer in a scientific study involving medical data collection from your records or information on ICU routine and treatments?				0.727
Yes	126/151 (83.4)	107/129 (82.9)	19/22 (86.4)	
Probably yes	14/151 (9.3)	13/129 (10.1)	1/22 (4.5)	
Probably no	2/151 (1.3)	2/129 (1.6)	0/22 (0.0)	
No	9/151 (6.0)	7/129 (5.4)	2/22 (9.1)	
Would you participate as a volunteer in a scientific study involving a new drug?				0.016
Yes	69/151 (45.7)	53/129 (41.1)	16/22 (72.7)	
Probably yes	27/151 (17.9)	25/129 (19.4)	2/22 (9.1)	
Probably no	27/151 (17.9)	23/129 (17.8)	4/22 (18.2)	
No	28/151 (18.5)	28/129 (21.7)	0/22 (0.0)	
Would you participate as a volunteer in a scientific study of a new surgical treatment?				0.152
Yes	51/150 (34.0)	40/128 (31.3)	11/22 (50.0)	
Probably yes	37/150 (24.6)	32/128 (25.5)	5/22 (22.7)	
Probably no	31/150 (20.7)	26/128 (20.3)	5/22 (22.7)	
No	31/150 (20.7)	30/128 (23.4)	1/22 (4.5)	
Would you trust a relative with the decision to be enrolled in a scientific study				0.727
Yes	88/151 (58.3)	75/129 (58.1)	13/22 (59.1)	
Probably yes	18/151 (11.9)	16/129 (12.4)	2/22 (9.1)	
Probably no	12/151 (7.9)	9/129 (7.0)	3/22 (13.6)	
No	33/151 (21.9)	29/129 (22.5)	4/22 (18.2)	

#: For variables with missing data, summary data are based on available cases. P values were calculated with chi-square test.

Approximately half of the patients [48.3% (72/149 patients) would not change their opinion, i.e., no longer accept to participate in a scientific study if their legal representative did not support their enrollment, while 50% (75/150 patients) would change their opinion, i.e., no longer accept to participate in a scientific study if their primary attending physician did not support their enrollment (Table 2). Patients are more likely to accept to participate as volunteers in a scientific study only when it involves data collection from their medical records [92.7% (140/151)] or when a new drug is being tested [63.6% (96/151)] rather than when a new surgical treatment is being studied [58.6% (88/150)] (Table 2).

Finally, most of the patients [90.7% (136/150)] who would accept to be enrolled in a scientific study as volunteers would like to be informed about the final result of the scientific study they had been enrolled in. The survey responses provided by patients that would not participate as volunteers in a scientific study are presented in S3 Table in S1 File.

### Patients’ knowledge about the importance of research

Ninety-two percent (185/201) of patients agreed that in order to develop new treatments, research involving human beings must be conducted (S4 Table in S1 File). While 92.8% of patients trust results obtained by research conducted in private hospitals, only 66.5% and 54.1%, respectively, believed in results obtained by public hospitals and by the pharmaceutical industry (S4 Table in S1 File).

### Patients’ characteristics and their willingness to participate as volunteers

In a multivariable logistic regression analysis, only patients age ≥65 years (OR, 0.34; 95%CI, 0.14 to 0.67; p = 0.002) were independently associated with a lower odds of a patient to participate as a volunteer in scientific research (Table 3).

**Table 3:** Multivariate logistic regression analysis addressing patients’ characteristics associated with their willingness to participate as volunteers in a scientific study.

Characteristics	OR	95% CI	P value
Age, years			
<65	Reference		
> = 65	0.34	0.17–0.67	0.002
Gender			
Female	Reference		
Male	1.94	0.99–3.79	0.054
Educational level			
Illiterate / primary school	Reference		
High school	1.01	0.30–3.44	0.991
Higher education / Master’s / PhD	0.67	0.23–2.00	0.476
Religion			
Others / None	Reference		
Catholic	1.00	0.49–2.05	0.988
Family income			
< = 10 minimum wages	Reference		
>10 minimum wages	0.95	0.44–2.05	0.907

Participants were pooled into two groups according to their willingness to participate as volunteers in a scientific study (Yes/Probably yes) and (No/Probably no). OR: odds ratio, 95% CI: 95% confidence interval. The multivariate model had an area (95%CI) under the receiver operating characteristic curve of 0.66 (0.58–0.74) and a Hosmer-Lemeshow chi-square of 5.570 (p = 0.591).

### Patients’ opinion about deferred consent

Out of 153 ICU patients willing to be enrolled in a scientific study as volunteers (Table 2), 70.2% (106/151) of patients would trust a relative with the decision to be enrolled in a scientific study (Table 2). The agreement between patients’ and surrogates’ opinion concerning participation as volunteers in a scientific study was poor [Kappa = 0.11 (IC95% -0.02 to 0.25); p = 0.071)] (S2 Table in S1 File).

If a consent for study participation had been obtained, 69.1% (103/149) of patients would continue participating in the study until its conclusion, 23.5% (35/149) would allow researchers to use data collected to date, but would withdraw from the study on that occasion, and 7.4% (11/149) would withdraw from the study on that occasion and would not allow researchers to use data collected to date.

### Legal representatives’ answers

Out of 208 legal representatives who answered the survey, 87% (181/208) of the participants would allow their relative to participate as volunteers in scientific research during their stay in the ICU (Fig 1 and S2 Table in S1 File).

Legal representatives were more prone to allowing their relative to participate as volunteers in scientific research when only data collected from their medical records was being tested than when a medical or surgical treatment was being tested (Table 4). Legal representatives at a public hospital were more prone to allowing their relatives to participate as volunteers in scientific research testing a new medical or surgical therapy compared to legal representatives at a private hospital (Table 4).

**Table 4:** Legal representatives’ answers to survey accordingly to the type of hospital. Data presented nº/total (%)^#^.

	All legal representatives (N = 208)	Private Hospital (N = 181)	Public Hospital (N = 27)	P value
Would you authorize your relative to participate as a volunteer in scientific research during their stay in the ICU?				0.510
Yes	127/208 (61.1)	107/181 (59.1)	20/27 (74.1)	
Probably yes	54/208 (26.0)	49/181 (27.1)	5/27 (18.5)	
Probably no	11/208 (5.3)	10/181 (5.5)	1/27 (3.7)	
No	16/208 (7.7)	15/181 (8.3)	1/27 (3.7)	
Would you authorize your relative to participate as a volunteer in scientific research involving only data collection from medical records and/or data comprising ICU care and treatment routine?				
Yes	139/190 (73.2)	116/164 (70.7)	23/26 (88.5)	0.282
Probably yes	39/190 (20.5)	37/164 (22.6)	2/26 (7.7)	
Probably no	10/190 (5.3)	9/164 (5.5)	1/26 (3.8)	
No	2/190 (1.1)	2/164 (1.2)	0/26 (0.0)	
Would you authorize your relative to participate as a volunteer in scientific research involving a new medication?				0.018
Yes	58/190 (30.5)	46/164 (28.0)	12/26 (46.2)	
Probably yes	66/190 (34.7)	54/164 (32.9)	12/26 (46.2)	
Probably no	47/190 (24.8)	45/164 (27.4)	2/26 (7.7)	
No	19/190 (10.0)	19/164 (11.6)	0/26 (0.0)	
Would you authorize your relative to participate as a volunteer in scientific research involving a new surgical treatment?				0.031
Yes	46/188 (24.5)	36/162 (22.2)	10/26 (38.5)	
Probably yes	72/188 (38.3)	59/162 (36.4)	13/26 (50.0)	
Probably no	52/188 (27.7)	50/162 (30.9)	2/26 (7.7)	
No	18/188 (9.6)	17/162 (10.5)	1/26 (3.8)	
In situations of emergency/urgency (loss of consciousness, cardiac arrest, etc) it is not possible to request authorization (consent) from patient or from legal representative about his/her participation in scientific research. If your relative did not regain consciousness and if you were informed that he/she was included in scientific research, would you?				0.452
Allow him/her to continue participating until research was concluded	119/188 (63.3)	100/162 (61.7)	19/26 (73.1)	
Allow only data collected to date to be used and would request his/her removal from research	52/188 (27.7)	46/162 (28.4)	6/26 (23.1)	
Request his/her immediate removal from research and would not allow use of any data collected	17/188 (9.0)	16/162 (9.9)	1/26 (3.8)	

#: For variables with missing data, summary data are based on available cases.

If a consent for study participation had been obtained and the respective relative did not regain consciousness, 63.3% (46/188) of legal representatives would allow their relative to continue participating in the study until its conclusion (Table 4).

## Discussion

In this multicenter survey conducted in two medical-surgical ICUs including 208 pairs of ICU patients and their respective legal representatives, we demonstrated that nearly three out of four ICU patients surveyed would like to be enrolled in a scientific study as volunteers. We also demonstrated that surrogates’ opinion poorly reflects patients’ opinion concerning enrollment in clinical research, since agreement between patients’ and legal authorized representatives’ opinion was poor.

The main purpose of informed consent in the context of clinical research is to ensure that ethical principles of autonomy and the self-determination of participating subjects are preserved [11, 12]. However, our findings support the hypothesis that informed consent obtained in emergency situations by surrogates may not fully reflect patients’ willingness to participate in scientific research as volunteers [13–17].

Similar findings were reported by Newman and cols. in a survey conducted with sixty-nine adult patients and surrogates admitted to a medical ICU in the United States [14]. The authors demonstrated a marked discrepancy between patients’ and surrogates’ willingness to participate as volunteers in scientific research [14]. More interestingly, discrepancy between patients and surrogates opinion increased as complexity of hypothetical studies increased, ranging from less than 5% in an observational study involving only demographic and clinical data collection up to approximately 50% in a randomized controlled clinical trial [14].

In another study conducted in ten ICUs in France, two hypothetical research studies with different levels of risk to patients were applied to patients and their surrogates on the day of ICU discharge to the wards [13]–one involving minimal risk (a hypothetical study comparing crystalloids to colloids for volume expansion in septic shock), and another, greater-than-minimal risk to patients (a hypothetical study comparing early vs. late tracheotomy in patients requiring invasive mechanical ventilation). The authors reported that surrogates’ opinion underestimated patients’ wishes concerning their willingness to participate in scientific research, with a discrepancy rate of 32% and 42% between patients and surrogates, respectively, for minimal risk and greater-than-minimal risk hypothetical studies [13].

This study found that the most common reason for ICU patients to consent to participate as volunteers in a scientific study was the fact that they believed that in the future other patients might benefit from the study results. Similar findings were reported by Mehta and cols., who studied 96 surrogates’ (substitute decision makers) motivations to provide consent or not for their critically ill adult family members to be enrolled in scientific research as volunteers [18]. The authors reported that the vast majority of substitute decision makers (91%) would agree with enrollment as they believe that the study results will help others in the future [18].

Our study has limitations. First, this survey was designed with predefined answers i.e., yes / probably yes / probably no / no, which might have compromised the judgement and perceptions of participants. Nevertheless, when compared to similar surveys in literature, it involves a remarkable number of responders [14] besides being the first survey carried out comparing the opinion of patients and their legal representatives in Latin America. Secondly, all surveys were applied by the study investigators; therefore, participants might have provided favorable answers, thus leading to bias. Thirdly, we excluded unconscious patients in this study. Thus, our results need to be interpreted with caution when considering more severe patients, whose legal representatives may have been experiencing severe emotional stress, which would compromise their decision making. Fourth, the survey was structured with a logical sequence of questions, which precluded bias minimization by randomizing or alternating the sequence of questions. Finally, most patients and their relatives were from a private hospital, which may limit the external validity of our results.

## Conclusion

The majority of patients admitted to the ICU were willing to be enrolled in a scientific study as volunteers, also after a deferred informed consent procedure has been used. Nevertheless, contradictory opinions between patients and their and their legal representatives’ concerning enrollment in a scientific study were often observed. Efforts should be made in order to improve knowledge of ICU patients and their relatives concerning the importance and value of medical research to improve patients’ engagement in clinical research.

## Supporting information

S1 File (DOCX)
