# Supplementary material for: Intensive care unit patients’ opinion on enrollment in clinical research: A multicenter survey
Source: PLoS One. 2020 Aug 13;15(8):e0236675. doi: 10.1371/journal.pone.0236675 (PMC7425869; doi:10.1371/journal.pone.0236675)
Supplement: S1 File — (DOCX) [file pone.0236675.s001.docx]

**critically ill patients’ and surrogates’ opinion on enrollment in clinical research: A MULTICENTER SURVEY**

**Supporting information**

### Flavia Julie do Amaral Pfeilsticker, Carolina Aguiar SantAnna Siqueri, Niklas Soderberg Campos, Fernanda Guimarães Aguiar, Maria Laura Romagnoli, Renato Carneiro de Freitas Chaves, Carolina Scoqui Guimarães, Adriano José Pereira, Ricardo Luiz Cordioli, Ary Serpa Neto, Murillo Santucci Cesar Assunção, Thiago Domingos Corrêa

**SURVEY FOR PATIENTS**

1. **DEMOGRAPHIC DATA**
2. Age: ____
3. Gender: ( ) Male ( ) Female
4. Educational level

( ) Higher education

( ) High school

( ) Primary school

( ) Master’s / PhD

( ) Illiterate

1. Religion

( ) Catholic

( ) Other

( ) Evangelic

( ) No religion

( ) Jewish

1. Place of residence

( ) Southeast

( ) South

( ) Central-west

( ) Northeast

( ) North

1. Family income

( ) > 10 minimum wages

( ) 2 - 5 minimum wages

( ) 6 - 10 minimum wages

( ) 1 minimum wage

( ) No income

1. **ON BEING ENROLLED IN A RESEARCH AS A VOLUNTEER**
2. Have you ever been enrolled in a scientific study?

( ) Yes

( ) No

1. Would you like to be enrolled in a scientific study as a volunteer?

( ) Yes (go to question 12)

( ) Probably yes (go to question 12)

( ) Probably no

( ) No

1. If the question is no or probably no, what is the reason?

( ) I’m afraid my condition gets worse

( ) I don’t trust the investigator

( ) I don’t want to serve as a “guinea pig”

( ) I prefer not to say the reason(s)

( ) I can’t explain

( ) I don’t know

1. Would you change your opinion, that is, would you participate in a scientific study if your legal relative supported your enrollment?

( ) Yes

( ) Probably yes

( ) Probably no

( ) No

1. Would you change your opinion, that is, would you participate in a scientific study if your physician supported your enrollment?

( ) Yes

( ) Probably yes

( ) Probably no

( ) No

1. Would you change your opinion, that is, would you participate in a scientific study if your legal representative did not support your enrollment?

( ) Yes

( ) Probably yes

( ) Probably no

( ) No

1. Would you change your opinion, that is, would you participate in a scientific study if your physician did not support your enrollment?

( ) Yes

( ) Probably yes

( ) Probably no

( ) No

1. Would you be enrolled as a volunteer in a scientific study for collection of medical data from your records or information on ICU routine and treatments?

( ) Yes

( ) Probably yes

( ) Probably no

( ) No

1. Would you participate as a volunteer in a scientific study of a drug? n (%)

( ) Yes

( ) Probably yes

( ) Probably no

( ) No

1. Would you participate as a volunteer in a scientific study of a new surgical treatment? n (%)

( ) Yes

( ) Probably yes

( ) Probably no

( ) No

1. Which option motivates you to participate in a scientific study? (You may choose as many alternatives as you like)

( ) The study offers benefits immediately/in the future.

( ) The study offers benefits for the general population in the future.

( ) The study offers financial profits for your participation

( ) Your physician’s request / recommendation.

( ) No treatment options.

1. Have you ever heard of informed consent?

( ) Yes

( ) No

1. Would you trust a relative with the decision of getting enrolled in a scientific study?

( ) Yes

( ) Probably yes

( ) Probably no

( ) No

1. In emergency situations (i.e. loss of consciousness, cardiac arrest), it is not possible to get an authorization from a relative or a legal representative for participation in scientific research. After recovering consciousness and being informed of your participation in scientific research, would you?

( ) Continue participating until its conclusion

( ) Allow use of data collected to date and ask to withdraw from the study

( ) Leave the study and not allow use of data from that moment on

1. Would you like to be informed about the final results of the scientific study you’ve been enrolled in?

( ) Yes

( ) Probably yes

( ) Probably no

( ) No

1. **ABOUT THE IMPORTANCE OF RESEARCH**
2. Do you agree that in order to discover effective treatments doctors should conduct research involving humans?

( ) Yes

( ) Probably yes

( ) Probably no

( ) No

1. Do you trust results obtained by a study conducted by a public hospital?

( ) Yes

( ) Probably yes

( ) Probably no

( ) No

1. Do you trust results obtained by a study conducted by a private hospital?

( ) Yes

( ) Probably yes

( ) Probably no

( ) No

1. Do you trust results obtained by a study conducted by the pharmaceutical industry?

( ) Yes

( ) Probably yes

( ) Probably no

( ) No

1. Do you trust results obtained by a study conducted by Hospital Israelita Albert Einstein?

( ) Yes

( ) Probably yes

( ) Probably no

( ) No

1. Do you trust results obtained by a study conducted by Hospital Municipal Vila Santa Catarina?

( ) Yes

( ) Probably yes

( ) Probably no

( ) No

1. In your opinion, how acceptable is it for a hospital to conduct scientific research involving human beings?

( ) Highly

( ) Moderately

( ) Poorly

( ) Not at all

( ) I don’t know

**SURVEY FOR LEGALLY AUTHORIZED REPRESENTATIVES**

1. **DEMOGRAPHIC DATA**
2. Age: ____
3. Gender: ( ) Male ( ) Female
4. Kinship degree

( ) Husband / wife

( ) Son / daughter

( ) Parent

( ) Brother / sister

( ) Other

1. Would you authorize your relative to participate as a volunteer in a scientific research during their stay in the ICU?

( ) Yes

( ) Probably yes

( ) Probably no

( ) No

1. **OPINION ON AUTHORIZING PATIENTS TO BE ENROLLED IN RESEARCH**
2. Would you authorize your relative to participate as a volunteer in a scientific research involving only collection of data from medical records and/or data on ICU care and treatment routine?

( ) Yes

( ) Probably yes

( ) Probably no

( ) No

1. Would you authorize your relative to participate as a volunteer in a scientific research involving a new medication?

( ) Yes

( ) Probably yes

( ) Probably no

( ) No

1. Would you authorize your relative to participate as a volunteer in a scientific research involving a new surgical treatment?

( ) Yes

( ) Probably yes

( ) Probably no

( ) No

1. In some situations of emergency/urgency (loss of
   consciousness, cardiac arrest, etc) it is not possible to request authorization (consent) from patients or from legal representatives about their participation in a scientific research. If your relative did not regain consciousness and if you were informed that he/she was included in a scientific research, would you?

( ) Allow him/her to continue to participate until the research is concluded

( ) Allow that only the data collected until that moment were utilized and would request his/her removal from the research

( ) Request his/her immediate removal from the research and would not allow the use of any data collected

**S1 Table.** Characteristics of study participants according to the type of hospital. Data presented as median (interquartile range) or nº/total (%)^#^.

| **Characteristics** | **Private Hospital**  **(N = 181)** | **Public Hospital**  **(N = 27)** | **P value** |
| --- | --- | --- | --- |
| Age, years | 60 (44-75) | 63 (42-74) | 0.734^a^ |
| Female, gender | 89/181 (49.2) | 13/27 (48.1) | 0.921^b^ |
| Educational level |  |  | <0.001^b^ |
| Master’s / PhD | 19/181 (10.5) | 2/27 (7.4) |  |
| Higher education | 111/181 (61.3) | 7/27 (25.9) |  |
| High school | 36/181 (19.9) | 4/27 (14.8) |  |
| Primary school | 15/181 (8.3) | 12/27 (44.4) |  |
| Illiterate | 0/181 (0.0) | 2/27 (7.4) |  |
| Religion |  |  |  |
| Catholic | 127/181 (70.2) | 18/27 (66.7) | 0.032^b^ |
| Other | 16/181 (8.8) | 4/27 (14.8) |  |
| Evangelic | 10/181 (5.5) | 5/27 (18.5) |  |
| No religion | 15/181 (8.3) | 0/27 (0.0) |  |
| Jewish | 13/181 (7.2) | 0/27 (0.0) |  |
| Place of residence |  |  |  |
| Southeast | 150/181 (82.9) | 23/27 (85.2) | 0.773^b^ |
| South | 14/181 (7.7) | 2/27 (7.4) |  |
| Central-west | 8/181 (4.4) | 2/27 (7.4) |  |
| Northeast | 5/181 (2.8) | 0/27 (0.0) |  |
| North | 4/181 (2.2) | 0/27 (0.0) |  |
| Family income |  |  | <0.001^b^ |
| >10 minimum wages | 130/172 (75.6) | 3/26 (11.5) |  |
| 6-10 minimum wages | 21/172 (12.2) | 3/26 (11.5) |  |
| 2-5 minimum wages | 16/172 (9.3) | 17/26 (65.4) |  |
| 1 minimum wage | 4/172 (2.3) | 3/26 (11.5) |  |
| No income | 1/172 (0.6) | 0/26 (0.0) |  |
| **Legal Representatives** |  |  |  |
| Age, years | 49 (38-60) | 47 (32-57) | 0.328^a^ |
| Female, gender | 120/181 (66.3) | 17/27 (63.0) | 0.733^b^ |
| Kinship degree |  |  | 0.692^b^ |
| Husband / wife | 67/181 (37.0) | 8/27 (29.6) |  |
| Son / daughter | 56/181 (30.9) | 12/27 (44.4) |  |
| Parent | 23/181 (12.7) | 2/27 (7.4) |  |
| Brother / sister | 13/181 (7.2) | 2/27 (7.4) |  |
| Other | 22/181 (12.2) | 3/27 (11.1) |  |

#: For variables with missing data, summary data are based on available cases. P values were calculated with (a): Mann-Whitney U test or (b) chi-square test.

**S2 Table.** Agreement between patients’ and surrogates’ opinion concerning participation as volunteers in a scientific study. Data presented nº/total (%).

| Would you like to be enrolled in a scientific study as a volunteer? |  |
| --- | --- |
| Yes / Probably yes | 153/208 (73.6) |
| No / Probably no | 55/208 (26.4) |
| Would you authorize your relative to participate as a volunteer in a scientific research during their stay in the ICU? |  |
| Yes / Probably yes | 181/208 (87.0) |
| No / Probably no | 27/208 (13.0) |

Kappa=0.11 (IC95% -0.02 to 0.25); p = 0.071

**S3 Table.** Patients who would not be enrolled in a scientific study (No / Probably no) as a volunteer. Data presented nº/total (%)^#^.

| Have you ever been enrolled in a scientific study? |  |
| --- | --- |
| Yes | 4/55 (7.3) |
| No | 51/55 (92.7) |
| Why would you not participate in a scientific study as a volunteer? |  |
| I don’t know | 0/55 (0.0) |
| I can’t explain | 26/55 (47.3) |
| I’m afraid my condition gets worse | 13/55 (23.6) |
| I don’t want to serve as a “guinea pig” | 8/55 (14.5) |
| I prefer not to say the reason(s) | 8/55 (14.5) |
| I don’t trust study investigators | 3 (5.5) |
| Would you change your opinion, that is, would you participate in a scientific study if your legal relative supported your enrollment? |  |
| Yes | 7/54 (13.0) |
| Probably yes | 4/54 (7.4) |
| Probably no | 11/54 (20.4) |
| No | 32/54 (59.2) |
| Would you change your opinion, that is, would you participate in a scientific study if your physician supported your enrollment? |  |
| Yes | 13 (24.5) |
| Probably yes | 11 (20.7) |
| Probably no | 10 (18.9) |
| No | 19 (35.9) |

#: For variables with missing data, summary data are based on available cases.

**S4 Table.** Assessment of patients’ knowledge about the importance of research accordingly to the type of hospital. Data presented nº/total (%)^#^.

|  | **All patients**  **(N = 208)** | **Private Hospital**  **(N = 181)** | **Public Hospital**  **(N = 27)** | **P value** |
| --- | --- | --- | --- | --- |
| Do you agree that in order to discover effective treatments doctors should conduct research involving humans? |  |  |  | 0.522 |
| Yes | 157/201 (78.1) | 133/174 (76.4) | 24/27 (88.9) |  |
| Probably yes | 28/201 (13.9) | 26/174 (14.9) | 2/27 (7.4) |  |
| Probably no | 3/201 (1.5) | 3/174 (1.7) | 0/27 (0.0) |  |
| No | 13/201 (6.5) | 12/174 (6.9) | 1/27 (3.7) |  |
| Do you trust results obtained by a study conducted by a public hospital? |  |  |  | <0.001 |
| Yes | 90/206 (43.7) | 72/179 (40.2) | 18/27 (66.7) |  |
| Probably yes | 47/206 (22.8) | 38/179 (21.2) | 9/27 (33.3) |  |
| Probably no | 22/206 (10.7) | 22/179 (12.3) | 0/27 (0.0) |  |
| No | 47/206 (22.8) | 47/179 (26.3) | 0/27 (0.0) |  |
| Do you trust results obtained by a study conducted by a private hospital? |  |  |  | 0.486 |
| Yes | 137/207 (66.2) | 118/180 (65.6) | 19/27 (70.4) |  |
| Probably yes | 55/207 (26.6) | 47/180 (26.1) | 8/27 (29.6) |  |
| Probably no | 4/207 (1.9) | 4/180 (2.2) | 0/27 (0.0) |  |
| No | 11/207 (5.3) | 11/180 (6.1) | 0/27 (0.0) |  |
| Do you trust results obtained by a study conducted by the pharmaceutical industry? |  |  |  | 0.543 |
| Yes | 68/207 (32.9) | 59/180 (32.8) | 9/27 (33.6) |  |
| Probably yes | 44/207 (21.2) | 38/180 (21.1) | 6/27 (22.2) |  |
| Probably no | 37/207 (17.9) | 30/180 (16.7) | 7/27 (25.9) |  |
| No | 58/207 (28.0) | 53/180 (29.4) | 5/27 (18.5) |  |
| In your opinion, how acceptable is it for a hospital to conduct scientific research involving human beings? |  |  |  | 0.864 |
| Highly | 90/206 (43.7) | 76/179 (42.5) | 14/27 (51.9) |  |
| Moderately | 82/206 (39.8) | 72/179 (40.2) | 10/27 (37.0) |  |
| Poorly | 16/206 (7.8) | 15/179 (8.4) | 1/27 (3.7) |  |
| Not at all | 10/206 (4.8) | 9/179 (5.0) | 1/27 (3.7) |  |
| Don’t know | 8/206 (3.9) | 7/179 (3.9) | 1/27 (3.7) |  |

#: For variables with missing data, summary data are based on available cases. P values were calculated with chi-square test.
