# Supplementary material for: Intensive care unit patients’ opinion on enrollment in clinical research: A multicenter survey
Source: PLoS One. 2020 Aug 13;15(8):e0236675. doi: 10.1371/journal.pone.0236675 (PMC7425869; doi:10.1371/journal.pone.0236675)
Supplement: Attachment — Submitted filename: Response_to_Reviewers.docx [file pone.0236675.s002.docx]

São Paulo, June 26^th^, 2020.

Response to Editor and Reviewers’ comments

PONE-D-20-10276: **critically ill patients’ and surrogates’ opinion on enrollment in clinical research: A MULTICENTER SURVEY**

Dear Dr. Andrew Carl Miller

Academic Editor PLOS ONE

We would like to express our full appreciation for giving us the opportunity to revise our manuscript. We believe that the reviewers made insightful suggestions that greatly helped to address critical unresolved issues to improve the manuscript. All the changes in the paper are highlighted in green in the revised version to indicate the revised portions of the manuscript. We expect that our manuscript can be now suitable for publication in PLOS ONE.

I remain at your disposal to clarify any pending point.

Yours sincerely,

On behalf of all the authors,

Thiago D. Correa

**Journal Requirements**

When submitting your revision, we need you to address these additional requirements.

1. Thank you for including your ethics statement:

"This prospective observational study was conducted in two ICUs located in two hospitals (private and public) in São Paulo, Brazil after obtaining institutional approval at each site (CAAE: 62100416.8.1001.0071). Written informed consent was obtained from each study participant." Please amend your current ethics statement to include the full name of the ethics committee/institutional review board(s) that approved your specific study. Once you have amended this statement in the Methods section of the manuscript, please add the same text to the “Ethics Statement” field of the submission form (via “Edit Submission”).

Response: Thank you for your suggestion. We added the following sentence to the “Ethics Statement” field of the submission form and to the revised manuscript (Page 5, line 37-38):” The Ethics Committee of Hospital Israelita Albert Einstein (CAAE: 62100416.8.1001.0071) and of Secretaria Municipal da Saúde de São Paulo – SMS/SP (Municipal Secretary of Health of São Paulo) (CAAE: 62100416.8.3001.0086) approved the study protocol.”.

3. We note that you have indicated that data from this study are available upon request. PLOS only allows data to be available upon request if there are legal or ethical restrictions on sharing data publicly. For information on unacceptable data access restrictions, please see <http://journals.plos.org/plosone/s/data-availability#loc-unacceptable-data-access-restrictions>. In your revised cover letter, please address the following prompts:

a) If there are ethical or legal restrictions on sharing a de-identified data set, please explain them in detail (e.g., data contain potentially identifying or sensitive patient information) and who has imposed them (e.g., an ethics committee). Please also provide contact information for a data access committee, ethics committee, or other institutional body to which data requests may be sent.

b) If there are no restrictions, please upload the minimal anonymized data set necessary to replicate your study findings as either Supporting Information files or to a stable, public repository and provide us with the relevant URLs, DOIs, or accession numbers.

Response: The study data bank has been deposited at Dryard (https://doi.org/10.5061/dryad.66t1g1k06).

**Reviewer #1:** Thank you for the opportunity to review this well-written manuscript.

Response: Thank you very much for your constructive comments and suggestions.

1. Please insert line numbers.

Response: Added.

2. Methods: following IRB statement, please state that the manuscript adheres to the STROBE guidelines and provide proper citation.

Response: Thank you for your suggestion. We added the following sentence to the revised manuscript (Page 5, line 41-42): This study was reported in accordance with The Strengthening the Reporting of Observational Studies in Epidemiology (STROBE) guidelines.” The corresponding reference was added (Page 15).

3. Were there any other restrictions on inclusion / exclusion? - Spoken language, - Literacy, - Pregnancy status, - Vulnerable populations (prisoners, developmental delay/mental retardation, minority groups, etc…).

Response: We thank the reviewer for his suggestion. All patients with preserved cognitive function were eligible for the study. There were no limitations concerning pregnancy status or other minority groups. The investigators helped Illiterate patients to answer the survey by reading the statements for them. We added the following statements to the revised manuscript (Page 5, Lines 46-49): “Patients under 18 years old, patients unable to understand or to speak Portuguese, patients with no legal representative during ICU stay, patients with impaired consciousness at the time of evaluation (i.e., sedated patients or patients with delirium), and patients with developmental delay were excluded.”

4. State how the survey was developed, including whether the usability and technical functionality of the electronic questionnaire had been tested before fielding the questionnaire.

Response: We thank the reviewer for his important suggestion. The questionnaire was developed by the authors (FJAP, RLC and TDC). Content validation was performed by a panel of senior researchers (AJP, ASN and MSCA). They critically revised the survey instrument and judged whether the instrument meets its goal. The panel of senior researchers sent the survey for the developers to make the necessary adjustments. These adjustments were performed and the revised survey was reassessed by the panel. After the third revision, content validity was supported by the panel. Face validity, i.e, whether the survey instrument appears to test what it is supposed to and questionnaire's psychometric properties were tested by all other co-authors and by a small group of volunteers (10 employers) in both ICUs. The study database was structured in the Research Electronic Data Capture (REDCap), hosted in a private and safe server at Hospital Israelita Albert Einstein, which was filled out by the investigators. This information has been added to the revised manuscript (Page 6, lines 52-60 and lines 71-73).

5. Please provide descriptions of face validity and content validity.

Response: This issue has been addressed in question 4.

6. How/where was the survey announced or advertised?

Response: Eligible patients were personally advised about the survey by one of the study investigators within the first 48 hours of ICU admission.

7. Were any incentives offered (eg, monetary, prizes, or non-monetary incentives such as an offer to provide the survey results)?

Response: We thank the reviewer for addressing this important issue. No financial incentives were offered for participants of this study. This statement was added to the revised manuscript (Page 6, line 73-74).

8. In what timeframe were the data collected?

Response: Data was collected between January 2017 and May 2018. This information was presented in the revised manuscript (Page 8, lines 111-113) as follows: " Between January 2017 and May 2018, 208 pairs of ICU patients and their respective legal representatives answered the survey [87.0% (181/208) private hospital and 13.0% (27/208) public hospital].”

9. To prevent biases items can be randomized or alternated. Please state if this was done.

Response: The survey was structured with a logical sequence of questions, which precluded randomizing or alternating sequence of questions. This issue was added as a study limitation of revised manuscript as follows (Page 13, lines 232-234): “Fourth, the survey was structured with a logical sequence of questions, which precluded minimization of bias by randomizing or alternating the sequence of questions.”

10. Where respondents were able to review and change their answers?

Response: We thank the reviewer for addressing this important issue.
Participants who accepted to participate in this study responded to a traditional paper-and-pencil questionnaire, which was then put into an opaque envelope to preserve their anonymity. Once responded, the answers were not able to be revised. This information was added to the revised manuscript (Page 6, line 70) as follows: “Once responded, the answers were not able to be revised”.

11. Please describe the participation rate.

Response: Unfortunately, we did not register the number of patients who were invited to participate of this study. Therefore, we are not able to provide the participation rate. We apologize for this inconvenience.

12. Were only completed questionnaires analyzed? What threshold was used to determine completion (100%, 90%, 75%, etc.)?

Response: Only questionnaires with 90% or more of completion were eligible for analysis. We added the following statement to the revised manuscript (Page 6, line 74): “A threshold of more than 90% of answers was used to determine completion of questionnaires.”

13. Indicate whether any methods such as weighting of items or propensity scores have been used to adjust for the non-representative sample; if so, please describe the methods.

Response: Thank you for your comment. We did not use any methods to adjust for the non-representative sample.

14. How was data normality determined?

Response: Normality was tested with the Kolmogorov-Smirnov test. This was added to the revised manuscript (Page 7, lines 90-92).

15. Overall, the population seemed willing to enroll in a research study despite little prior exposure or knowledge of informed consent. It would be interesting to know what the willingness to participate was amongst those persons who DID have prior exposure / knowledge of informed consent.

Response: We appreciate the reviewer’s suggestion. Out of 208 ICU patients who answered the survey, 13.9% (29/208) had never been enrolled in a scientific study while 86.1% (179/208) had never been enrolled in a scientific study as volunteers. The willingness to be enrolled in a scientific study as volunteers did not differ between patients that already had been enrolled in a scientific study as a volunteer compared to those patients that had never been enrolled in a scientific study [86.2% (25/29) vs. 71.5% (128/179), respectively, p=0.096]. We added these additional results to the revised manuscript (Pages 8-9, lines 119-124).
